# Percutaneous Retrograde Technique Using Intramedullary Headless Compression Screws for Metacarpal Fractures Under Wide-Awake Local Anaesthesia No Tourniquet

**DOI:** 10.7759/cureus.31517

**Published:** 2022-11-14

**Authors:** Naufal Ahmed, Rory Norris, Aghna Faiaz, Anirudh Sharma, Santosh Bindumadhavan

**Affiliations:** 1 Trauma and Orthopaedics, Peterborough City Hospital, Peterborough, GBR; 2 Anaesthesia, Manipal Academy of Higher Education, Mangalore, IND

**Keywords:** walant, compression screw, retrograde technique, percutaneous, metacarpals

## Abstract

Background

The common modality of treatment of metacarpal fractures is nonsurgical. There are, however, a subset of patients and fracture types that require surgical correction, but surgery comes with its own problems like stiffness and scarring. Therefore, surgical operations must be minimally invasive barring complications of anaesthesia and the procedure. Therefore, we conducted this study to assess patient outcomes following treatment with percutaneous intramedullary screw fixation via the wide-awake local anaesthesia no tourniquet (WALANT) approach for unstable metacarpal fractures.

Methodology

We retrospectively analysed the records of 21 patients who received metacarpal fixations with headless compression screws at two district general hospitals in the United Kingdom from 2018 to 2020. We used wide-awake anaesthesia with 10 mL (1% lidocaine and 1 mL 8.4% sodium bicarbonate as a buffer) infiltrated around the superficial tissues on the dorsal aspect of the metacarpal bone, including the periosteum. The Jahss manoeuvre was used to reduce the fracture under the guidance of a mini C-arm. All patients had 3-mm Medartis cannulated compression screws (Medartis AG, Basel, Switzerland) (self-tapping) inserted retroactively using a 5-mm skin incision. The range of movement of the metacarpophalangeal joint was checked intraoperatively and shown to the patient for optimal postoperative rehabilitation. Patients underwent a two-week follow-up wound check and examination for pain (using the visual analogue scale (VAS)) or stiffness requiring physiotherapy. We used the Manchester-modified (M2) disability of the arm, shoulder, and hand (DASH) score to scrutinize the fracture union and the functional outcome of the hand. We also assessed the time to return to work.

Results

The study included 18 men and two women with a mean age of 22.6 years (range, 18 to 40). The fifth (n=16), fourth (n=4), and second metacarpals (n=1) were involved, and we saw transverse (n=10) and short oblique (n=11) fractures. Fractures healed in five weeks (range, four to six weeks). The mean M2 DASH score was 0.8 (range, 0 to 6), and mean total active motion was 240° (range, 230° to 260°). At the final follow-up, the mean extensor lag for the metacarpophalangeal joint was 5° (range, 0° to 15°), 7° for the proximal interphalangeal joint (range, 0° to 15°), and no lag at the distal interphalangeal joint. The average VAS score at the end of two weeks was 8/10 (range, 7 to 9). The average time for the return to daily activities was 2.56 weeks. We found no intraoperative complications in any of the patients. All patients went home on the same day postoperatively and gave feedback that their experience with WALANT was good to excellent. All patients had a good range of motion at the two-week follow-up, and the mean time to return to normal work was two to three weeks. The M2 DASH score measured was satisfactory.

Conclusions

This retrospective study assessed patient outcomes following treatment with percutaneous intramedullary screw fixation via the WALANT approach for unstable metacarpal fractures. WALANT was a quick and reliable alternative to fix unstable metacarpal fractures, especially for high-demand patients requiring a short recovery period before returning to regular activity. Further research with a larger sample size and a longer follow-up to analyse the outcome is warranted before an actual guideline can be established.

## Introduction

Metacarpal fractures constitute approximately 18% of all upper limb fractures below the elbow [1] and are the third most common fractures of the upper extremity, following the distal radius and phalangeal fractures [1,2]. The boxer's fracture (at the fifth metacarpal neck) is the most common of these, comprising 10% of all hand fractures [3]. There are various options to treat these fractures, and the most common treatment modality is nonoperative management, like splinting and plaster immobilization [4]. Lumbricals and interossei provide good coverage to the metacarpal bones, making them more stable than the phalanges. Treating these fractures depends on the patient's age and occupation, hand dominance, degree of rotational deformity, level and type of fracture, and degree of displacement. Operative methods include closed or open reduction using Kirschner wires (K-wires), screws, plates, and intramedullary compression screws. Complications from surgical interventions include stiffness, proximal interphalangeal joint fixed flexion contracture, and extensor lag [5].

Plate and screw fixation provides stabilization and an early range of motion and is useful in comminuted fractures but has mixed clinical results (extensive scarring and stiffness) [5]. The conventional K-wire technique is accepted everywhere but has a higher complication rate of 15% to 18% [4,5]. These include the chance of infection (7%), loosening/migration of the pin tract (5%), nonunion (4%), and immobilization of the patient with splints or plasters for several weeks [6,7]. However, the retrograde intramedullary screw fixation technique has gained popularity in recent years as it avoids the need for immobilization and prevents scarring and stiffness because it does not involve opening the fracture site [8,9].

Retrograde intramedullary screw fixation is done under wide-awake local anaesthesia no tourniquet (WALANT), which uses lidocaine with adrenaline instead of only adrenaline. Hemostasis provided by adrenaline around the operating site lasts for a few hours, even after surgery, preventing hematoma formation. Intraoperatively, it eliminates the need for a tourniquet, reducing operating time and ischemic pain that appears 15 to 20 minutes after application. WALANT also eliminates the need for nerve blocks, meaning that neurovascular structures are less likely to be damaged. The surgery can be performed in an upright position, which avoids respiratory compromise in susceptible patients. During the pandemic, WALANT aided in limiting unnecessary exposure to patients and staff preoperatively (because no preoperative evaluation is required) and postoperatively (because it can be done as a day-case surgery with early discharge). Adequate analgesia is provided by this technique, which helps avoid typical complications of regional or general anaesthesia, such as nausea and vomiting.

Since there is no conventional postoperative splinting or immobilization [10], this technique is useful in younger age-group professionals and skilled workers needing to get back to work as soon as possible. Stiffness and scarring with various surgical treatment options for metacarpal fractures have made subnormal functions acceptable outcomes in these fractures. Therefore, we conducted this study to assess patient outcomes following percutaneous intramedullary screw fixation via the WALANT approach for unstable metacarpal fractures.

An early abstract of this study was previously presented at the 2021 British Orthoapaedic Association Meeting, September 21, 2020.

## Materials and methods

We retrospectively analyzed 21 metacarpal fixation patients who received surgery with headless compression screws at two district general hospitals (Hinchingbrooke hospital, Huntingdon and Peterborough City hospital, Peterborough) in the United Kingdom from 2018 to 2020. All metacarpal fracture patients were first triaged; undisplaced fractures (especially the Boxer's fractures) were treated with a Bedford splint or buddy strap and then discharged with a safety netting. The displaced fractures were referred to the fracture clinic for the consultant to decide on conservative or operative treatment.

The inclusion criteria used for fixation with headless screws depended on the type of fracture: transverse two-part fractures that were typical for this technique and short oblique fractures. They also depended on the level of fracture (neck of the metacarpal and shaft were the common levels encountered), amount of angulation > 60° to 70° for metacarpal neck and > 30° for proximal and midshaft metacarpal fractures, shortening > 5 mm, and rotational deformity. Patients who tended to use their hands for fine motor movements, such as cyclists, musicians, doctors, nurses, and students, were included as their time to return to work was significantly shortened [10]. Patients were excluded if they were skeletally immature and had comminuted displaced fractures with more than three parts, long oblique fractures (which are more suited for open reduction and internal fixation with plates), open fractures, or fractures with minimal angulation (which was reduced in the emergency department/clinic with local anaesthesia and found to be in an acceptable position).

Preoperative

Qualifying patients who consented to receive surgery in the clinic under WALANT received the procedure. As the name implies, it is a surgery where patients are fully awake, and no tourniquet is applied. On the day of the surgery, the patients were given a local anaesthetic (1% lidocaine with 1:200,000 adrenaline) injected with a 27-gauge needle and one dose of intravenous (IV) antibiotic half an hour before the procedure. Every 10 mL of lidocaine was buffered with 1 mL of 8.4 % sodium bicarbonate to neutralize its pH, reducing the pain on injection and increasing the onset speed. The point of injection was over the dorsum of the hand from the base up until the head of the metacarpal, resulting in vasoconstriction and anaesthesia around that region, slowly reaching the periosteum of the bone. Anaesthetics were also supplemented around the hand's palmar aspect and the metacarpophalangeal joint (MCPJ) over the digital nerves. Health care providers used small needles (27 or 30-gauge), pinched the skin as the needle was inserted, and injected only the anaesthetized skin (except for the first time). They also inserted the needle perpendicular to the skin to avoid injecting the dermis, stabilized the needle tip using both hands, injected the first 2 mL slowly, waited 15 seconds, and then moved the needle tip. They injected proximal to distal, proceeded slowly, and asked for patient feedback often.

Intraoperative

The surgeons measured the metacarpal bone preoperatively to ensure the selection of the proper screw size by measuring the entire width of the bone and the narrowest width of the medullary canal isthmus, which are different for each finger. The surgeons ensured the screw was long enough to sustain the excessive forces and reduce the torsional forces on the fracture site [8]. They reduced the fracture (checked by mini C-arm) by flexing the metacarpophalangeal (MCP) and proximal joint to 90° and pushing the proximal phalanx towards the metacarpal head using the Jahss manoeuvre [11]. They marked an entry point percutaneously over the dorso-ulnar/radial aspect of the MCPJ using a guidewire and a 5-mm incision along the entry point. They passed a guidewire with the help of an image intensifier from the distal to the proximal end of the fracture and used an entry reamer to widen the entry point. A 3-mm Medartis self-tapping partially threaded screw (Medartis AG, Basel, Switzerland) was used to stabilize the fracture site.

The surgeon confirmed the burial of the headless screw inside the MCPJ by checking the image intensifier and then asked the patient (who was awake) to bend their fingers, while the team checked the rotations and cascade of the hand. They closed the wound with 3.0 Vicryl Rapide sutures (Ethicon Inc., Raritan, New Jersey, United States) after a thorough wash. Jelonet (Smith & Nephew plc, London, United Kingdom) and Cosmopore (Hartmann, Rock Hill, South Carolina, United States) dressings were applied, followed by a Bedford splint. The patient was discharged home with a two-week follow-up.

**Figure 1:**
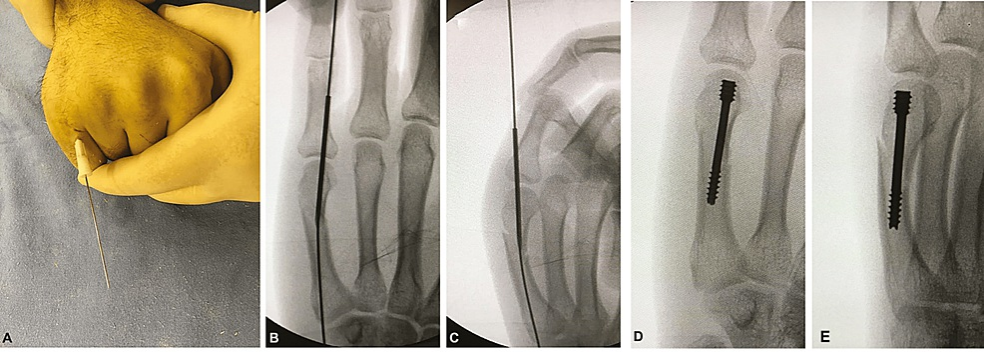
(A) The entry point for the guidewire with a 16-gauge needle technique. (B and C) Intraoperative anteroposterior (B) and oblique (C) views showing the reduction after passing the guidewire. (D and E) Intraoperative images showing the position of the screws and to check whether they are buried inside the joint.

To make the entry point easy in certain fractures, surgeons used the needle technique as shown in Figure 1, described by Celso et al. [4], to reduce metacarpal neck fractures. This involved positioning a 16-gauge needle to make an entry point at the centre of the metacarpal head, the position of which was checked with the image intensifier in both anteroposterior and lateral views. The needle was manually inserted into the medullary canal until it crossed the proximal part of the fracture, which was used as a sleeve for the guidewire. After the introduction of the guidewire, the needle was removed, and then a stab incision was made for the percutaneous entry of the 3-mm self-tapping screw.

Postoperative

Patients were advised to avoid lifting heavy weights and were encouraged to bend their fingers as far as pain permitted actively. No x-rays were taken, and we referred to images saved from the mini C-arm. During follow-up, the wounds were checked, and the patients' pain levels were assessed using the visual analogue scale (VAS). We also checked the range of motion in the MCPJs and the interphalangeal joints and evaluated x-rays.

A six- and 12-week review was conducted to scrutinize the fracture union and the functional outcome of the hand using the Manchester-modified (M2) disability of the arm, shoulder, and hand (DASH) questionnaire. We also recorded the time to return to work.

## Results

The sample included 20 patients (18 men, two women) with a mean age of 22.6 years (range, 18 to 40 years). The fifth (n=16), fourth (n=4), and second metacarpals (n=1) were involved, and we saw transverse (n=10) and short oblique (n=11) fractures. The mechanisms causing the fractures included punching against an object/person (n=10), falls (n=8), and injury from road traffic crashes (n=2). The sites of metacarpal fractures were the neck (n=10), proximal shaft (n=5), and mid-shaft (n=6).

The mean time from fracture to surgery was 14 ± 6 days. Fractures healed in five weeks (range, four to six weeks; Table 1). The mean M2 DASH score was 0.8 (range, 0 to 6), and mean total active motion was 240° (range, 230° to 260°). At the final follow-up, the mean extensor lag for the MCPJ was 5° (range, 0° to 15°), 7° for the proximal interphalangeal joint (range, 0° to 15°), and no lag at the distal interphalangeal joint. The average VAS score at the end of two weeks was 8/10 (range, 7 to 9). The average time for the return to daily activities was 2.56 weeks. Most patients were students and were right-hand dominant (n=16, left n=4), but a few were teachers and IT professionals.

**Table 1:** Postoperative clinical outcomes M2 DASH, Manchester-modified disabilities of arm, shoulder, and hand score; TAM, total active motion; MCPJ, metacarpophalangeal joint; PIPJ, proximal interphalangeal joint; DIPJ, distal interphalangeal joint; VAS, visual analogue scale.

Clinical Outcome	Results
Union at 6 weeks	100%
M2 DASH score in the postoperative period	0.8 (range, 0-6)
TAM in the postoperative period	240° (range, 230°-260°)
Average extensor lag at MCPJ at the last follow-up	5% (0%-15%)
Extensor lag at PIPJ at the last follow-up	7% (0%-15%)
Extensor lag at DIPJ at the last follow-up	0%
VAS score at two weeks	8/10 (range, 7-9)
Average time to return to work	2.56 weeks

There were no intraoperative complications. All patients went home on the same day and reported good to excellent feedback (which was obtained via a feedback form rating their overall experience between 1 and 5; 1 being very dissatisfied and 5 being very satisfied. All patients gave a rating between 4 and 5). One patient asked to be put to sleep to avoid hearing background theatre noise. He was sedated with a remifentanil infusion which kept him conscious and able to communicate but negated his anxiety. All patients were seen in the clinic at the end of two weeks for wound check, and the wounds healed well without complications. They were directly referred to the hand therapist during that visit. There were no loss of fracture reduction, implant breakage, infection, malrotation, chronic pain regional syndrome, or screw migration at the 12-week postoperative follow-up.

## Discussion

Metacarpal fractures are more forgiving in terms of management, as they can accept more angulation than phalangeal fractures because of their attachment with intermetacarpal ligaments and interossei [4]. The boxer's fracture also accepts more angulation than other metacarpal fractures [10]. In this study, we noticed that fractures that did well with intramedullary fixation were transverse and short oblique fractures (<2 times the shaft diameter) of the metacarpal shaft. The long oblique fractures (>2 times the shaft diameter) and comminuted fractures are at risk of fracture displacement and shortening [12]. This percutaneous retrograde intramedullary fixation for metacarpal and phalangeal fractures has gained popularity in the last decade, and the results are promising [13-18].

Our results aligned with those studies' outcomes. However, our study was unique due to the inclusion of two different hospitals in the United Kingdom, the use of WALANT, and the use of a mini C-arm operated by the surgeons themselves, avoiding the need for extra personnel. This was particularly useful during the pandemic. The WALANT technique reduced perioperative time and cost. Furthermore, physicians can obtain real-time patient feedback by getting them to bend their fingers and checking for malrotations. The percutaneous entry point for the guidewire and screw has been disputed. Some authors have performed a mini-open technique involving careful cutting and suturing the extensor mechanism back into place. However, we used a percutaneous entry approach backed by results of Urbanschitz et al. [19], where the mini-open and percutaneous techniques were compared for the insertion of headless screws, and the respective amount of tendon damage in both these techniques in cadavers was compared. Although the amount of tendon damage was greater in the percutaneous technique (25% to 49%), it did not cross the threshold (>50%), requiring an extensor tendon repair.

The subsequent controversy regarding the use of a retrograde percutaneous technique is the concern of damaging the MCPJ articular cartilage. Urbanschitz et al. suggested that the amount of cartilage damage is approximately 5% to 6% for all fingers [19]. Moreover, the MCPJ is not a weight-bearing joint without axial loading. The MCPJ is round and congruous with the proximal phalanx, and our entry point is slightly dorsal without involving the articular surface. However, this approach is not supported by long-term clinical trials, and further studies are necessary to assess osteoarthritis development due to this practice.

To support the practice of immediate mobilisation of the fingers postfixation, few biomechanical studies show that the load to failure was equal to or higher than K-wires but less than plates/screws [13]. This shows that it is safe to mobilize these patients immediately without splints/plasters. The other advantage of this fixation is its usefulness in multiple metacarpal and phalangeal fractures in early mobilisation and rehabilitation [8].

Our study has several important limitations. This was a retrospective study with a small sample size without randomisation. No similar techniques were compared, and no long-term follow-up was performed. Therefore, possible complications such as implant failure and arthritis could not be gauged. Although the overall efficacy of all the metacarpal fractures seemed to be good, we cannot comment upon the individual efficacy of each fracture pattern due to the small study size and lack of control group comparisons. Implant removal is important to consider and evaluate further; removal is more complex than the initial procedure. Also, there is a possibility of screw breakage warranting a removal, which is equally challenging. The procedure also requires appropriate surgical skills and appropriately sized screws for the canal.

## Conclusions

This study assessed patient outcomes following treatment with percutaneous intramedullary screw fixation via the WALANT approach for unstable metacarpal fractures. Retrograde intramedullary fixation of metacarpal fractures with compression screws showed good clinical and radiological results in all patients. WALANT was a quick and reliable approach to fixing unstable metacarpal fractures, especially for high-demand patients requiring a short recovery period before returning to normal activity. As this study has proven positive results, additional multicentric trials with longer follow-up monitoring are warranted. Also, future studies should use a control group for comparison, as following a minimally invasive technique could lead to quicker rehabilitation.

## References

[1] Chung KC, Spilson SV (2001). The frequency and epidemiology of hand and forearm fractures in the United States. J Hand Surg Am.

[2] Court-Brown CM, Caesar B (2006). Epidemiology of adult fractures: a review. Injury.

[3] Malik S, Herron T, Rosenberg N (2022). Fifth metacarpal fractures. StatPearls [Internet].

[4] Folberg CR, Alves JA, Cadore OP, Sirena FM (2021). Osteosynthesis of fractures of the metacarpal neck with self-compressing screw - preliminary analysis of 21 cases. Rev Bras Ortop (Sao Paulo).

[5] Page SM, Stern PJ (1998). Complications and range of motion following plate fixation of metacarpal and phalangeal fractures. J Hand Surg Am.

[6] Botte MJ, Davis JLW, Rose BA, von Schroeder HP, Gellman H, Zinberg EM, Abrams RA (1992). Complication of smooth pin fixation of fracture and dislocation in the hand and wrist. Clin Orthop Relat Res.

[7] Stahl S, Schwartz O (2001). Complications of K-wire fixation of fractures and dislocations in the hand and wrist. Arch Orthop Trauma Surg.

[8] Chao J, Patel A, Shah A (2021). Intramedullary screw fixation comprehensive technique guide for metacarpal and phalanx fractures: pearls and pitfalls. Plast Reconstr Surg Glob Open.

[9] Esteban-Feliu I, Gallardo-Calero I, Barrera-Ochoa S, Lluch-Bergadà A, Alabau-Rodriguez S, Mir-Bulló X (2021). Analysis of 3 different operative techniques for extra-articular fractures of the phalanges and metacarpals. Hand (N Y).

[10] Poggetti A, Nucci AM, Giesen T, Calcagni M, Marchetti S, Lisanti M (2018). Percutaneous intramedullary headless screw fixation and wide-awake anesthesia to treat metacarpal fractures: early results in 25 patients. J Hand Microsurg.

[11] Boulton CL, Salzler M, Mudgal CS (2010). Intramedullary cannulated headless screw fixation of a comminuted subcapital metacarpal fracture: case report. J Hand Surg Am.

[12] Bloom JM, Hammert WC (2014). Evidence-based medicine: metacarpal fractures. Plast Reconstr Surg.

[13] Morway GR, Rider T, Jones CM (2021). Retrograde intramedullary screw fixation for metacarpal fractures: a systematic review. Hand (NY).

[14] Guidi M, Frueh FS, Besmens I, Calcagni M (2020). Intramedullary compression screw fixation of metacarpal and phalangeal fractures. EFORT Open Rev.

[15] Warrender WJ, Ruchelsman DE, Livesey MG, Mudgal CS, Rivlin M (2020). Low rate of complications following intramedullary headless compression screw fixation of metacarpal fractures. Hand (N Y).

[16] Eisenberg G, Clain JB, Feinberg-Zadek N, Leibman M, Belsky M, Ruchelsman DE (2020). Clinical outcomes of limited open intramedullary headless screw fixation of metacarpal fractures in 91 consecutive patients. Hand (N Y).

[17] Siddiqui AA, Kumar J, Jamil M, Adeel M, Kaimkhani GM (2019). Fixation of metacarpal fractures using intramedullary headless compression screws: a tertiary care institution experience. Cureus.

[18] Beck CM, Horesh E, Taub PJ (2019). Intramedullary screw fixation of metacarpal fractures results in excellent functional outcomes: a literature review. Plast Reconstr Surg.

[19] Urbanschitz L, Dreu M, Wagner J, Kaufmann R, Jeserschek JM, Borbas P (2020). Cartilage and extensor tendon defects after headless compression screw fixation of phalangeal and metacarpal fractures. J Hand Surg Eur Vol.

